# Interpreting the results of early skin tests after perioperative anaphylaxis requires special attention: a case report and review of literature

**DOI:** 10.1007/s00540-020-02802-x

**Published:** 2020-05-31

**Authors:** Masaki Orihara, Tomonori Takazawa, Kazuhiro Nagumo, Shinya Sakamoto, Tatsuo Horiuchi, Shigeru Saito

**Affiliations:** 1 Department of Anesthesiology, Gunma University Graduate School of Medicine, 3-39-22, Showa-machi, Maebashi, 371-8511 Japan; 2 Intensive Care Unit, Gunma University Hospital, 3-39-15, Showa-machi, Maebashi, 371-8511 Japan

**Keywords:** Anaphylaxis, Rocuronium, Skin test, Basophil activation test

## Abstract

Skin tests are the gold standard for detecting the culprit drug of anaphylaxis, and should ideally be performed after an interval of 4–6 weeks after the reaction to avoid false-negative results. However, when re-operation cannot be delayed and early allergy tests are necessary, special attention is required during subsequent anesthesia, because early skin tests tend to produce false-negative results. This report presents a case of rocuronium-induced anaphylaxis in which early skin tests showed negative results for all the drugs tested. The second anesthesia was safely performed by avoiding all the drugs used for the first anesthesia. Ultimately, skin tests and basophil activation tests (BATs) performed after re-operation demonstrated rocuronium as the drug responsible for anaphylaxis. We recommend performing BATs in addition to skin tests to improve the accuracy of diagnosis of anaphylaxis. In this report, we also discuss interpretation of the results of early skin tests and subsequent selection of drugs for anesthesia. After postponement of surgery due to anaphylaxis, we are often required to perform allergy tests at an early stage if re-operation cannot be delayed. In such cases, skin test results alone should not be used to guide subsequent anesthesia management to avoid recurrent anaphylaxis.

## Introduction

Anaphylaxis is defined as a life-threatening systemic hypersensitivity reaction of sudden onset [1]. After the occurrence of anaphylaxis, allergologic assessment is essential to identify the causative agent and prevent recurrences [2]. Skin tests remain the gold standard for detection of the culprit drug, and should ideally be performed at an interval of 4–6 weeks after the reaction [2, 3]. However, in some cases, surgery cannot be delayed for this long a period, and investigation must be done earlier. While recent guidelines suggest that skin tests can be performed immediately after a reaction [4, 5], several articles warn about the possibility of false-negative results, especially with early skin tests [6]. Although subsequent uneventful anesthesia is guaranteed as long as true-negative drugs are used, the use of drugs that are false negative in skin tests could result in a second severe anaphylactic reaction [7, 8]. Thus, even if early skin test results are negative for all the drugs tested, special attention is required during the subsequent anesthesia.

Here, we describe a case of anaphylaxis in which early skin tests showed negative results for all the drugs tested, despite which all of these drugs were avoided during subsequent anesthesia and surgery. As a result, the surgery was successfully completed without recurrence of anaphylaxis.

## Case report

This case was part of a prospective observational study approved by the institutional review board of Gunma University Hospital (ID: 1084) and registered with the University Hospital Medical Information Network Clinical Trials Registry (ID: 000022365). Written informed consent was obtained from the patient’s parent prior to the tests.

A 19-year-old, 82-kg, 175-cm male was scheduled for open reduction and internal fixation for mandibular fracture. He had no past history of allergy or surgery. Anesthesia was induced with a 3-μg/ml target-controlled infusion of propofol and 80-mg rocuronium. At the same time, continuous administration of remifentanil was started at the rate of 0.3 μg/kg/min. Immediately after anesthesia induction, he demonstrated skin erythema and wheals mainly in the anterior chest area. Although endotracheal intubation was successfully performed, his oxygen saturation decreased to approximately 80%, together with the development of wheezing. The patient did not show any cardiovascular symptoms. Suspecting a possible allergic reaction or asthmatic attack, he was treated with 200-μg salbutamol inhalation and intravenous injection of 6.6-mg dexamethasone, with subsequent improvement in respiratory symptoms. The surgery was, however, canceled and he was transferred to the intensive care unit, where he remained until his trachea was extubated 3 h after the event.

Since early surgery was necessary to prevent occlusal deficiency and trismus, surgery was rescheduled for 10 days after the event. Both skin prick tests (SPTs) and intradermal tests (IDTs) using all the drugs suspected to be the possible cause of anaphylaxis were performed eight days after the event, all of which showed negative reactions. Skin tests should ideally be performed a minimum of 4–6 weeks after the reaction to avoid false-negative results [9]. Since we considered the possibility of false-negative results in skin tests, our second general anesthesia plan did not include any of the suspected offending drugs. The second anesthesia was induced with bolus intravenous injection of 12-mg midazolam and 30-mg pentazocine, and inhalation of 8% sevoflurane and 50% nitrous oxide. After successful endotracheal intubation, anesthesia was maintained with 2% sevoflurane and 60% nitrous oxide. The operation was successfully completed within 231 min. The patient’s postoperative course was uneventful and he was discharged home 14 days later.

**Fig. 1:**
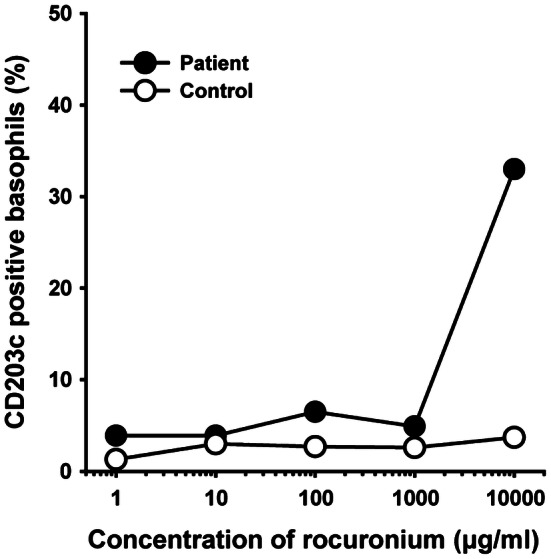
Results of basophil activation tests. CD203c upregulation was evident in the patient with rocuronium-induced anaphylaxis (closed circles) as compared to a control individual (open circles)

Seven weeks after the episode, skin tests were performed with propofol, rocuronium and remifentanil. While SPTs showed a negative reaction to all the drugs tested, IDTs showed a positive reaction to only 50-μg/ml rocuronium (Table 1). We also performed basophil activation tests (BATs) on the same day as the skin tests, detailed methods for which are described elsewhere [10–12]. Briefly, CD203c was used as a marker to detect activated basophils using a flow cytometer (FACSCanto II; BD Biosciences, San Jose, CA). The patient’s ratio of activated basophils was calculated and compared with that of a healthy male volunteer with no allergic skin test reaction to rocuronium. We confirmed a marked increase in the rate of activated basophils after stimulation with 10,000-μg/ml rocuronium. No such increase was observed after stimulation with any of the concentrations of rocuronium examined in the control individual (Fig. 1).

**Table 1:** Results of skin tests

	First skin tests at 8 days after the reaction				Second skin tests at 7 weeks after the reaction			
Drug	SPT	IDT			SPT	IDT		
			Wheal (mm)	Flare (mm)			Wheal (mm)	Flare (mm)
Saline	−	−			−	−		
Histamine	+	+	8	12	+	+	10	30
Propofol	−	−			−	−		
Rocuronium	−	−			−	+	6	10
Remifentanil	−	−			−	−		

Drug concentrations for SPTs: histamine 10 mg/ml, propofol 0.1, 1, 10 mg/ml, rocuronium 0.1, 1, 10 mg/ml, remifentanil 0.5, 5, 50 μg/ml

Drug concentrations for IDTs: histamine 10 μg/ml, propofol 0.01, 0.1, 1 mg/ml, rocuronium 0.5, 5, 50 μg/ml, remifentanil 0.05, 0.5, 5 μg/ml

*SPT* skin prick test; *IDT* intradermal test

## Discussion

We report here a case of rocuronium-induced anaphylaxis in which the second anesthesia was safely performed by avoiding all the drugs used for the first anesthesia. Ultimately, skin tests and BATs performed after re-operation demonstrated that rocuronium was the drug responsible for anaphylaxis.

Skin tests are widely used to determine the culprit drug for an anaphylactic reaction during anesthesia. In general, skin tests allow accurate diagnosis of drug hypersensitivity in more than 70% of cases [13]. As already described, experts recommend performing skin tests 4–6 weeks after the reaction [2, 3, 9].

Early allergy studies could be important when surgery is urgent. Often, surgery is discontinued or postponed in patients who develop a severe allergic reaction during anesthesia. When the surgery cannot be delayed, such as in patients with aggressive tumors or those who require emergency surgery, performing allergy tests soon after the event is inevitable [6, 14]. In our case, due to the urgent need for surgery, we had to perform early skin tests. However, since we anticipated that the early skin test results would be equivocal, our second general anesthesia plan did not include any of the suspected offending drugs to prevent recurrence. When skin tests are carried out earlier than the recommended 4–6 weeks after anaphylaxis, the risk of false-negative results cannot be ruled out. Indeed, in a review of previous literature, we found five cases in which the first skin tests performed within three weeks of the reaction were negative for all culprit drugs tested [6, 15–17] (Table 2). In three of the five cases, surgery was suspended until second skin tests were performed at least six weeks after the reaction to identify the offending drug.

**Table 2:** Case reports in which the first skin tests performed within three weeks showed negative results for all suspected drugs

Case number	Age	Sex	Procedure	Culprit drug	First skin tests		Second skin tests		Outcome of subsequent surgery	Reference number
					Delay	Results	Delay	Results		
1	42	F	Hysterectomy for menorrhagia	d-tubocurarine	4 days	IDT −	3 weeks	IDT 1:1000 +	Not described	15
2	44	M	Cervical discectomy and fusion for cervical myelopathy and progressive bilateral lower extremity weakness	Cefazolin	6 days	SPT −, IDT −	6 weeks	SPT 1 mg/ml +	Un eventful*	16
3	71	M	Revision of a knee prosthesis	Chlorhexidine	6 days	SPT −	11 weeks	SPT 1:1 + , IDT 1:10,000 +	Uneventful*	6
4	58	F	Intravaginal ultrasound examination for vaginal bleeding	Natural rubber latex	12 days	SPT −	3 months	SPT +	Not described	17
5	71	M	Hemicolectomy for cecal carcinoma	Rocuronium	20 days	SPT slightly suspicious, IDT −	6 weeks and 5 days	SPT 1:1 + , IDT 1:100 +	Uneventful*	6

*F* female; *M* male; *SPT* skin prick test; *IDT* intradermal test

^*^After the first surgery was postponed, the subsequent surgery could be completed without any problem by avoiding the drugs that showed positive results in the second skin tests

On the other hand, the causative drug was successfully identified by early skin tests in several cases [6, 18–23] (Table 3). In seven cases, skin tests performed between one and 14 days after the anaphylactic reaction showed positive results. Although the patient’s outcome following re-operation was not described in one case, re-operation was successfully completed in the remaining six cases by avoiding the drug that tested positive in early skin tests. Further, in two of the cases who subsequently underwent a second skin test, test results were consistent with those of the first skin tests (Table 3).

**Table 3:** Case reports in which early skin tests showed positive results to any of the drugs tested

Case number	Age	Sex	Procedure	Culprit drug	First skin tests		Subsequent surgery		Second skin tests		Reference number
					Delay	Results	Delay	Outcome	Delay	Results	
1	17	F	Cholecystectomy	Rocuronium	1 day	IDT 1:1000 +	2 days	Uneventful†	10 weeks	Skin testing +	18
2	52	M	Temporal lobectomy for treatment of poorly controlled epilepsy	Chlorhexidine	2 days	SPT 0.2% + , IDT 1:1000 +	Not described	Uneventful†	ND		19
3	58	F	Resection of rectal cancer	Rocuronium	3 days	IDT 1:1000 +	7 days	Uneventful†	ND		20
4	68	M	Mediastinoscopy to stage a carcinoma of the lung	Rocuronium	4 days	SPT 1:1 +	7 days	Uneventful†	ND		6
5	59	M	Resection of rectal cancer	Cisatracurium	7 days	IDT 0.02 mg/ml +	10 days	Uneventful†	6 weeks	SPT 2 mg/ml +	21
6	53	F	Excision of breast cancer	Isosulfan blue	10 days	SPT +	Not described		ND		22
7	70	M	Partial nephrectomy	Cisatracurium	14 days	SPT 1:1000 +	3 months	Uneventful†	ND		23

Case 1: Since the patient was soon to have final school exams, her family desired that her illness be resolved as quickly as possible

Case 7: The surgery was changed to total nephrectomy after the first skin tests

*F* female; *M* male; *SPT* skin prick test; *IDT* intradermal test; *ND* tests not performed

^†^After the first surgery was postponed, the subsequent surgery could be completed without any problem by avoiding the drugs that showed positive results on the first skin tests

Taken together, waiting for 4–6 weeks in accordance with the guidelines might not be possible in cases that require a rapid diagnosis. If early skin tests show positive reactions to any of the culprit drugs, avoiding these drugs during subsequent anesthesia would be acceptable. Conversely, no previous reports have described safe second anesthesia using the suspected drug(s) when all the drugs tested showed negative reactions in early skin tests. Thus, as far as possible, all suspected drugs should be avoided in such situations.

We performed BATs in addition to skin tests after re-operation. Although skin tests remain the gold standard for detection of the culprit drug, the positive predictive value of skin tests is not 100%. Hence, there seems to be room for other tests, including BATs. Since BATs are in vitro tests, the risks and burden on patients are minimal. Given the high specificity of BATs for neuromuscular blocking agents (NMBAs) (between 81 and 100% [24]), the combination of BATs and skin tests would allow diagnosis of anaphylaxis with high accuracy [25].

A pivotal factor in interpreting the results of skin tests is the cross-reactivity between drugs. We did not use any NMBAs for the second anesthesia, because rocuronium exhibits cross-reactivity with alternative NMBAs [26, 27]. For example, an Australian study demonstrated that patients with rocuronium anaphylaxis were skin test positive to succinylcholine (44%) and vecuronium (40%) [26]. Since there are only three NMBAs currently available in Japan, we do not have much of a choice for safer alternatives if anaphylaxis occurs with any of these NMBAs. Indeed, the second skin tests showed that rocuronium was the cause of anaphylaxis in our case. If this patient needs to be anesthetized in future, the only option would be to not use an NMBA. This is a common problem faced by Japanese anesthesiologists. The introduction of benzylisoquinoliniums, such as cisatracurium, in Japan is expected, because they are reported to have low cross-reactivity with rocuronium (5%) [26]. In fact, safe use of benzylisoquinoliniums in patients with rocuronium-induced anaphylaxis has been reported in a country where these drugs are available [28].

After postponement of surgery due to anaphylaxis, we are often required to perform allergy tests at an early stage when re-operation cannot be delayed. In such cases, skin test results alone should not be used to guide subsequent anesthesia management to avoid recurrent anaphylaxis.
